# Does smoking cessation affect postoperative healing following oral surgery among smokers? – a systematic review

**DOI:** 10.1186/s12903-024-03989-1

**Published:** 2024-02-15

**Authors:** Magnus Olsson, Eva Nordendahl, Björn Klinge, Anders Ekbom, Christoffer Edlund, Michael Fored, Johan Sundström, Aron Naimi-Akbar

**Affiliations:** 1 Faculty of Odontology, Health Technology Assessment-Odontology (HTA-O), Malmö University, Malmö, SE-205 06 Sweden; 2 Clinical Epidemiology Division, Dept of Medicine, Karolinska Institutet, Solna, Sweden; 3 Department of Medical Sciences, Clinical Epidemiology, Uppsala University, Uppsala, Sweden; 4 Department of Oral Surgery, Sunderby Hospital, Luleå, Sweden; 5 Department of Periodontology, Eastman Institute, Folktandvården Stockholm AB, Stockholm, Sweden; 6 Periodontology and Dental Prophylaxis Unit, Department of Dental Medicine, Karolinska Institutet, Huddinge, Sweden

**Keywords:** Smoking cessation, Oral surgery, Complication, Healing, Guideline

## Abstract

**Background:**

It is well documented that smokers suffer increased risk of postoperative complications after medical surgery, for example delayed healing and increased risk of infection. It is also known that preoperative smoking cessation can reduce the risk of these complications. Because of this there are guidelines regarding preoperative smoking cessation in non-oral medical surgery. There are however no specific guidelines regarding oral surgical procedures, such as surgical extractions, dentoalveolar surgery, periodontal surgery, or dental implantation. Nevertheless, it is common that dentists and oral surgeons recommend smoking cessation pre to oral surgical procedures. The aim with this systematic review was to see if there are any evidence in the literature, supporting preoperative smoking cessation in oral surgical procedures.

**Methods:**

A systematic search of the electronic databases PubMed, Scopus, Web of Science, and Cochrane was conducted to identify studies addressing the effect of preoperative smoking cessation in oral surgical procedures. Included publications were subjected to preidentified inclusion criterion. Six examiners performed the eligibility and quality assessment of relevant studies. Risk of bias was assessed using ROBINS-I and RoB 2. Certainty assessment was carried out using GRADE.

**Results:**

The initial search resulted in 2255 records, and after removal of 148 duplicates, 16 articles met an acceptable level of relevance. These were read in full text, whereof 12 articles were excluded, due to different intervention, outcome, or study design than stated in the review protocol. One study remained with moderate risk of bias and three were excluded due to high risk of bias.

**Conclusion:**

This systematic review could not determine the effect of smoking cessation pre to oral surgical procedures, in smokers. This indicates lack of knowledge in the effects of smoking cessation. We also conclude a lack of knowledge in how to design smoking cessation in the most effective way.

## Background

Tobacco usage is a significant contributor to preventable morbidity and mortality and tobacco currently accounts for around six million deaths annually. This number is expected to increase to eight million by 2030 [1, 2]. Tobacco smoking harms nearly every organ in the body [3]. Smoked tobacco also increases the risk for postoperative complications after medical surgery. Studies among smokers have shown that healing after surgery is delayed and that there is an increased risk for postoperative infections [4]. A suggested explanation to this is that smoked tobacco affects oxygen supply to the tissues, altering the healing process which increases the risk for infections [5]. By extension, this leads to increased medical expenses and suffering compared to non-smoking patients [3]. Studies also show that smoking cessation before surgery can lower the risk for the above complications. Patient counseling, nicotine replacement therapy or a combination of both, are examples of cessation regimens used before surgical treatment. Examples of counseling are, shorter intervention and intensive smoking cessation intervention [3, 6]. In short, evidence in the literature, supporting smoking cessation before medical surgery, is well supported [5, 7, 8]. However, when it comes to oral surgical procedures, such as, surgical extractions, dentoalveolar surgery, periodontal surgery, or dental implantation, evidence is scarce. Nevertheless, it is common that patients are recommended smoking cessation before oral surgical procedures. Therefore, the aim of this systematic review is to investigate if there is any evidence present in the literature, supporting preoperative smoking cessation, in oral surgical procedures. And, furthermore, how the smoking cessation should be designed in the best suitable way. For example, the duration of the cessation and when to introduce it pre to oral surgical procedures, in smokers.

## Methods

### Protocol and registration

This systematic review was conducted, following the Preferred Reporting Items for Systematic Reviews and Meta-Analyses (PRISMA), [9]. Based on the aim with this review, a PROSPERO review protocol was developed (PROSPERO ID: CRD42021282952). This was done prior to the data collection process and no amendments to the protocol were done during the study process. PROSPERO is an international database of systematic review protocols. The protocol acts as a guideline during the review process and minimizes the risk of ad hoc modification. It contains information regarding, review title, review question, search terms, data sources and screening criteria [10].

### Eligibility criteria

In this review, smokers undergoing oral surgical procedures with smoking cessation, were compared to smokers undergoing oral surgical procedures without smoking cessation. To be included in the review, the following inclusion and exclusion criteria had to be fulfilled. Cohort studies, randomized controlled trials, and controlled clinical trials, regarding oral surgery, written in English, were included. Publications regarding cancer treatments or biopsies were excluded. Year of publication was not taken in consideration.

### Information sources

The initial literature search and search strategies were conducted together with an information specialist at the Malmö University library in Sweden. The literature search was performed within the following database sources, PubMed (Medline), The Cochrane Library (Wiley), Web of Science (Clarivate) and Scopus (Elsevier), to identify records addressing the effect of preoperative smoking cessation in oral surgical procedures. All identified records were subjected to the above pre-identified criteria, followed by an analysis using the Population Intervention Control Outcome system (PICO) (Table 1). The search was conducted on the same date for all databases, 2023-05-25. Hand search was conducted by searching through the references of the four articles assessed for bias (Fig. 2). We searched the grey literature using the query: “oral surgery” AND “smoking cessation” on Google Scholar (2024-01-16), and the first 60 results were screened. A complete list of the search strategies can be found in Table 2.

**Table 1:** Data extraction

Criteria	Description
*Research question*	Does smoking cessation affect postoperative healing following surgery in the oral cavity among smokers?
*Population*	Smoking patients who will receive surgery in the oral cavity
*Intervention*	Smoking cessation
*Control*	No cessation or different regimen for smoking cessation
*Outcome*	Postoperative infection
	Complications
	Pain
	Health related quality of life
*Inclusion criteria*	Cohort studies
	Controlled clinical trials
	Publication available in full text
*Exclusion criteria*	Publications in languages other than English
	Publications regarding cancer treatments or biopsies

**Table 2:** Search strategies. All searches conducted 2023-05-25

Database	Search terms	References found
*PubMed (Medline)*	#1: Periodontal Surgery OR Mucogingival Surgery OR Periodontal Reconstructive Surgery OR Periodontal Regenerative Surgery OR Implant Periodontal Surgery OR Dentoalveolar Surgery OR Surgical Extraction OR Dental Surgery OR Oral Surgery OR Oral Surgical OR Dental Implantation OR Tooth Extraction OR “Implant-Supported Dental Prosthesis” OR “Implant Supported Dental Prosthesis” OR Peri-Implantitis OR “Peri Implantitis” OR “Surgery, Oral”[Mesh] OR “Oral Surgical Procedures”[Mesh] OR “Dental Implantation”[Mesh] OR “Tooth Extraction”[Mesh] OR “Peri-Implantitis”[Mesh] OR “Dental Prosthesis, Implant-Supported”[Mesh]	441,291
	#2: Smoking Cessation OR Giving Up Smoking OR Quitting Smoking OR Stopping Smoking OR “Smoking Cessation”[Mesh]	48,825
	#3: 1 AND 2	392
*Cochrane Library*	#1: “Periodontal Surgery” OR “Mucogingival Surgery” OR “Periodontal Reconstructive Surgery” OR “Periodontal Regenerative Surgery” OR “Implant Periodontal Surgery” OR “Dentoalveolar Surgery” OR “Surgical Extraction” OR “Dental Surgery” OR “Oral Surgery” OR “Oral Surgical” OR “Dental Implantation” OR “Tooth Extraction” OR “Implant-Supported Dental Prosthesis” OR “Implant Supported Dental Prosthesis” OR Peri-Implantitis OR “Peri Implantitis” OR [mh “Surgery, Oral”] OR [mh “Oral Surgical Procedures”] OR [mh “Dental Implantation”] OR [mh “Tooth Extraction”] OR [mh Peri-Implantitis] OR [mh “Dental Prosthesis, Implant-Supported”]	21,216
	#2: “Smoking Cessation” OR “Giving Up Smoking” OR “Quitting Smoking” OR “Stopping Smoking” OR [mh “Smoking Cessation”]	9804
	#3: 1 AND 2	16
*Web of Science*	#1: ALL(“Periodontal Surgery” OR “Mucogingival Surgery” OR “Periodontal Reconstructive Surgery” OR “Periodontal Regenerative Surgery” OR “Implant Periodontal Surgery” OR “Dentoalveolar Surgery” OR “Surgical Extraction” OR “Dental Surgery” OR “Oral Surgery” OR “Oral Surgical” OR “Surgery, Oral” OR “Oral Surgical Procedures” OR “Dental Implantation, Subperiosteal” OR “Tooth Extraction” OR Peri-Implantitis/Complications OR Peri-Implantitis/surgery OR “Dental Prosthesis, Implant-Supported”)	41,484
	#2: “Smoking Cessation” OR “Giving Up Smoking” OR “Quitting Smoking” OR “Stopping Smoking”	38,004
	#3: 1 AND 2	17
*Scopus*	#1: “Periodontal Surgery” OR “Mucogingival Surgery” OR “Periodontal Reconstructive Surgery” OR “Periodontal Regenerative Surgery” OR “Implant Periodontal Surgery” OR “Dentoalveolar Surgery” OR “Surgical Extraction” OR “Dental Surgery” OR “Oral Surgery” OR “Oral Surgical” OR “Dental Implantation” OR “Tooth Extraction” OR “Implant-Supported Dental Prosthesis” OR “Implant Supported Dental Prosthesis” OR Peri-Implantitis OR “Peri Implantitis” OR “Surgery, Oral” OR “Oral Surgical Procedures” OR “Dental Implantation” OR “Tooth Extraction” OR Peri-Implantitis OR “Dental Prosthesis, Implant-Supported”	357,865
	#2: “Smoking Cessation” OR “Giving Up Smoking” OR “Quitting Smoking” OR “Stopping Smoking”	145,044
	#3: 1 AND 2	1531

*MeSH* Medical Subject Headings, used to index articles in the National Library of Medicine

### Study selection

Six examiners were divided into two groups, (BK, EN, CE, and ANA, AE, MO). This was done to share the workload of the first step in the screening process. Disagreement was solved through discussion with all six examiners. In all other steps both groups performed the eligibility assessment of relevant studies and consensus was reached in cases where disagreement occurred. The online collaboration tool Rayyan for systematic reviews was used in all steps during the selection process [11].

### Assessment of risk of bias

All included studies were assessed for bias using Risk Of Bias In Non-randomised Studies - of Interventions (ROBINS-I) [12]. Pre-defined confounding factors were socioeconomic status, education, income, comorbidity, sex, and age. In the data extraction process, the intervention and information about, study population, study-design, outcomes, and effect measures, was performed by MO and controlled by ANA (Fig. 2). Randomized clinical trials were planned to be assessed for bias using the risk-of-bias tool for randomized trials (RoB 2), however no such studies were included [13]. Reporting bias assessment was planned but not applicable due to lack of studies.

### Certainty assessment

We assessed certainty of evidence with the Grading of Recommendations, Assessments, Development, and Evaluation (GRADE) [14].

## Results

### Study selection

**Fig. 1:**
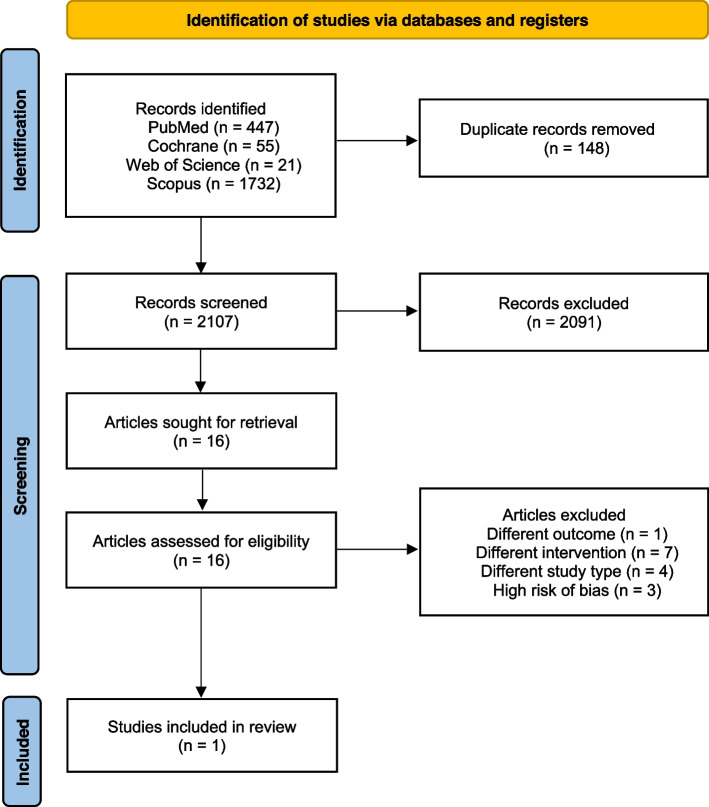
PRISMA flowchart

As stated before, this review was conducted, following the PRISMA guidelines [9]. The initial search resulted in 2255 records, and after removal of 148 duplicates, 16 articles met an acceptable level of relevance. These were read in full text, whereof 12 articles were excluded, due to different intervention, outcome, or study design than stated in the PROSPERO protocol. All steps in the study selection process are shown in the PRISMA flowchart (Fig. 1). For some of the studies more than one exclusion criterion was met, however, only one of the exclusion criteria for each study is presented in Table 3. Grey literature searches did not render any new references to include.

**Table 3:** Excluded publications after full-text evaluation and reasons for exclusion (*n* = 12)

*Reason for exclusion: intervention*	
Qiao F, et al. 2020. A Validated Model to Predict Postoperative Symptom Severity After Mandibular Third Molar Removal. Journal of Oral and Maxillofacial Surgery. 2020;78 (6):893–901.	
Windael S, et al. 2020. The Long-Term Effect of Smoking on 10 Years’ Survival and Success of Dental Implants: A Prospective Analysis of 453 Implants in a Non-University Setting. J Clin Med.9 (4).	
Al Amri MD, et al. 2017. Comparison of peri-implant soft tissue parameters and crestal bone loss around immediately loaded and delayed loaded implants in smokers and non-smokers: 5-Year follow-up results. Journal of Periodontology. 2017;88 (1):3–9.	
Nakayama Y et al. 2020. A multicenter prospective cohort study on the effect of smoking cessation on periodontal therapies in Japan. J Oral Sci.63 (1):114–8.	
Holliday R et al. 2019. A feasibility study with embedded pilot randomised controlled trial and process evaluation of electronic cigarettes for smoking cessation in patients with periodontitis. Pilot Feasibility Stud. 2019;5:74.	
Sella A, et al. 2020. Evaluation of surgical treatment of oroantral fistulae in smokers versus non-smokers. Medicina (Lithuania). 2020;56 (6):1–13.	
de Oliveira-Neto OB, et al. 2018. Risk of bias assessment of systematic reviews regarding dental implant placement in smokers: An umbrella systematic review. Journal of Prosthetic Dentistry. 2018;120 (2):198–203.	
*Reason for exclusion: outcome*	
Nagao T, et al. 2021. A multicentre tobacco cessation intervention study in the dental setting in Japan. Int Dent J.	
*Reason for exclusion: study design*	
Needleman I, et al. 2006. Evaluation of tobacco use cessation (TUC) counselling in the dental office. Oral Health and Preventive Dentistry. 2006;4 (1):27–47.	
Song F, et al. 2015. Identifying and recruiting smokers for preoperative smoking cessation--a systematic review of methods reported in published studies. Syst Rev.4:157.	
Kanmaz M, et al. 2021.. Periodontal treatment outcomes in smokers: A narrative review. Tobacco Induced Diseases. 2021;19.	
Zaki A, et al. 2018. Interventions in the preoperative clinic for long term smoking cessation: a quantitative systematic review. Can J Anaesth.55 (1):11–21.	

### Risk of bias

**Fig. 2:**
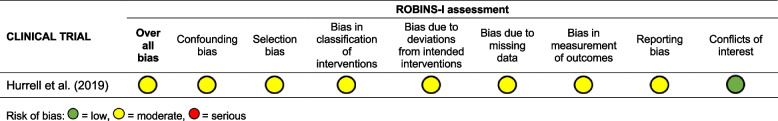
Methodological assessment of the remaining articles after full text assessment (*n* = 4) with the risk of bias in non-randomized studies of interventions (ROBINS-I) tool. One study was estimated to have a moderate risk of overall bias and was included. Three studies [15–17] were removed without further review because the risk of confounding bias was considered critical

The four remaining articles were assessed for bias using ROBINS-I [12]. Three articles were removed in the first domain due to critical risk of confounding bias [15–17], these are summarised in Table 4. In contrast to these, one study handled confounding bias using multivariate, logistic regression and assessed as moderate risk of overall bias, Hurrell et al. 2019 (Fig. 2) [18].

**Table 4:** Studies with unacceptable high risk of confounding bias

Author, year, country	Study design	Study population	Intervention	Control	Outcome	Comment
**Bain, 1996, Scotland **[15]	Prospective longitudinal study	Patients receiving dental implants	Smoking cessation	No smoking or smoking	Implant failure	Not handling confounding factors
**Mundt et al, 2006, Germany **[17]	Retrospective cohort study	Patients receiving dental implants	Former smoker	Current smoker	Implant failure	Not handling confounding factors, in relation to our research question
**Levin et al, 2004, Israel **[16]	Retrospective cohort study	Patients receiving onlay bone grafts or sinus lifts	Former smoker	No-smoking	Complications after surgery	Not handling confounding factors

### Included study

Only one study was included in the review, Hurrell et al. 2019 [18], Patient compliance and mandible fractures: a prospective study. This study investigated factors associated with patient compliance and how compliance affected the treatment outcome in mandible fractures, in the oral and maxillofacial unit, at a tertiary hospital in Australia. Smoking cessation was one of the compliance factors investigated. Smokers were instructed to refrain from smoking and the compliance was recorded as yes or no during the follow-up. Only 16% out of 215 patients were compliant to smoking cessation and the factor was not associated with treatment outcome. A complete summary of the smoking cessation intervention is shown in Table 5.

**Table 5:** Data extraction

*Author, year, country*	Hurrell et al. 2019 Australia [18]			
*Study type*	Cohort			
*Study period*	18 months (January 27, 2014 - July 26, 2015)			
*Population*	n: 215			
	Gender(m/f): 181/34			
	Mean age: 31			
	Smokers: 53%			
	Smoking cessation compliance: 16%			
	Mandible fractures: 359			
	Transoral ORIF: 90%			
	Extraoral ORIF: 7%			
	IMF in isolation: 3%			
	Single fracture: 44%			
	Two fractures: 47%			
	Three or more fractures: 9%			
	Comminuted fractures: 11%			
	Tooth present in fracture: 77%			
*Intervention*	Smoking cessation compliance y/n			
*Outcome*	Infection, dehiscence, non-union,			
	hardware exposure, nerve damage,			
	trismus, return to theatre			
*Results*		*OR*	*95% CI*	*P-value*
	Infection	0.53	0.06–4-91	0.57
	Dehiscence	0.44	0.01–14-99	0.65
	Non-union	0.42	0.01–31-98	0.69
	Hardware exposure	0.67	0.02–22-75	0.82
	Nerve damage	1.58	0.43–5.83	0.50
	Trismus	2.95	0.60–14.50	0.18
	Return to theatre	2.89	0.38–21.97	0.24

### Certainty assessment

In the included study, smoking cessation compliance was one of the interventions investigated and the primary outcomes were infection, dehiscence, non-union, hardware exposure, nerve damage, trismus and return to theatre, as summarized in Table 5. The pre-identified outcomes of interest for this review, postoperative infection, complications, pain, and health related quality of life are stated in the PICO (Table 1). As only one study was included, a meta-analysis was not applicable. However, the investigated outcomes would all have come out as, very low, following the GRADE guidelines. The confidence intervals are wide for all outcomes and the research question can therefore not be answered with any confidence.

## Discussion

This systematic review was conducted with the aim to investigate present literature for evidence, supporting preoperative smoking cessation, in oral surgical procedures. However, no such evidence could be found, indicating lack of knowledge in this area. In the final step, only one study was included. This study was conducted in the oral and maxillofacial unit, at a tertiary hospital where most patients came via the emergency department. This setting made it very difficult to put patients on preoperative smoking cessation. Despite this the study fulfilled all inclusion criteria and was therefore included in this review. Several of the studies in this systematic review were excluded due to that smokers were compared to non-smokers, instead of the effect of smoking cessation among smokers. As stated earlier, smoking is harmful and affects almost every organ in the body [3]. Smoking cessation has proven to affect the outcome of non-oral medical surgical procedures in a positive way [4, 8, 19]. Previous studies have also shown that information about the risks with tobacco to patients undergoing surgery could help them quit smoking [19, 20]. There are many well documented, positive effects from smoking cessation, and it is already recommended, for example in the Swedish national guidelines for dentistry, provided by The National Board of Health and Welfare, in Sweden [21]. Furthermore, the risk in recommending smoking cessation is probably non-existent. Could it be that there already is consensus supporting smoking cessation? This might be one of several reasons to why the number of studies in the field of oral surgical procedures are scarce. One other possible explanation could be that it is generally difficult to study tobacco habits due to lack of reliable registry data and the fact that smoking data is self-reported. There are some biomarker tests available, making it possible for researchers to evaluate compliance, and efforts have been made how to classify smoking cessation duration in a more standardized way [22, 23]. It is important to point out that the lack of specific studies on smoking cessation and its effect on oral surgical procedure outcome, in no way indicates that smoking cessation should not be recommended in these procedures. On the contrary, it would be interesting to see if there are even more benefits from smoking cessation, or risk with smoking that we are unaware of today. Contributing with knowledge that could motivate patients to quit smoking and help oral surgeons and dentists in their daily work. Out of the four studies that were controlled for bias, only the included study handled bias in an acceptable way. Randomized clinical trials or other larger epidemiological studies on smoking cessation, where confounders have been taken in account, are needed to obtain more reliable and generalizable results. The strength of this review was that the PRISMA guidelines and the recommended steps when conducting a systematic review, were followed, and an information specialist to assist with our search was consulted. The tools RAYYAN and ROBINS-I, were used and no amendments were made during the process. As in all systematic reviews, the limitation is dependent on the research material available. In this case the material was scarce. Only English publications were included and from the date of the search until publication, no new search was conducted. The overall ambition was to contribute with more research revealing the positive effects of smoking cessation and help smokers to quit smoking. More research in this field is needed.

## Conclusion

This systematic review could not determine the effect of smoking cessation pre to oral surgical procedures, in smokers. This indicates lack of knowledge in the effects of smoking cessation in this field. We also conclude a lack of knowledge in how to design smoking cessation in the most effective way in this area.

## Data Availability

All data generated or analyzed during this study are included in this published article.

## Abbreviations

PRISMA: Preferred Reporting Items for Systematic Reviews and Meta-Analyses
PICO: Population, Intervention, Control, Outcome
GRADE: Grading of Recommendations, Assessments, Development, and Evaluation
RoB 2: Risk-of-bias tool for randomized trials
ROBINS-I: Risk Of Bias In Non-randomised Studies - of Interventions
